# In the Qaidam Basin, Soil Nutrients Directly or Indirectly Affect Desert Ecosystem Stability under Drought Stress through Plant Nutrients

**DOI:** 10.3390/plants13131849

**Published:** 2024-07-05

**Authors:** Yunhao Zhao, Hui Chen, Hongyan Sun, Fan Yang

**Affiliations:** Hebei Key Laboratory of Environmental Change and Ecological Construction, Hebei Technology Innovation Center for Remote Sensing Identification of Environmental Change, School of Geographical Sciences, Hebei Normal University, Shijiazhuang 050024, China; zhaoyunhao@stu.hebtu.edu.cn (Y.Z.); shyan19990321@163.com (H.S.); geoyf@stu.hebtu.edu.cn (F.Y.)

**Keywords:** desert ecosystem, Qaidam Basin, drought gradient, soil and plant nutrients, ecosystem stability

## Abstract

The low nutrient content of soil in desert ecosystems results in unique physiological and ecological characteristics of plants under long-term water and nutrient stress, which is the basis for the productivity and stability maintenance of the desert ecosystem. However, the relationship between the soil and the plant nutrient elements in the desert ecosystem and its mechanism for maintaining ecosystem stability is still unclear. In this study, 35 sampling sites were established in an area with typical desert vegetation in the Qaidam Basin, based on a drought gradient. A total of 90 soil samples and 100 plant samples were collected, and the soil’s physico-chemical properties, as well as the nutrient elements in the plant leaves, were measured. Regression analysis, redundancy analysis (RDA), the Theil–Sen Median and Mann–Kendall methods, the structural equation model (SEM), and other methods were employed to analyze the distribution characteristics of the soil and plant nutrient elements along the drought gradient and the relationship between the soil and leaf nutrient elements and its impact on ecosystem stability. The results provided the following conclusions: Compared with the nutrient elements in plant leaves, the soil’s nutrient elements had a more obvious regularity of distribution along the drought gradient. A strong correlation was observed between the soil and leaf nutrient elements, with soil organic carbon and alkali-hydrolyzed nitrogen identified as important factors influencing the leaf nutrient content. The SEM showed that the soil’s organic carbon had a positive effect on ecosystem stability by influencing the leaf carbon, while the soil’s available phosphorus and the mean annual temperature had a direct positive effect on stability, and the soil’s total nitrogen had a negative effect on stability. In general, the soil nutrient content was high in areas with a low mean annual temperature and high precipitation, and the ecosystem stability in the area distribution of typical desert vegetation in the Qaidam Basin was low. These findings reveal that soil nutrients affect the stability of desert ecosystems directly or indirectly through plant nutrients in the Qaidam Basin, which is crucial for maintaining the stability of desert ecosystems with the background of climate change.

## 1. Introduction

The world’s drylands, including dryland ecosystems such as deserts or degraded lands, grasslands, savannahs, and much of the Mediterranean, account for about 41% of the global land area and are expected to warm faster than the rest of the world, triggering more intense droughts [1,2]. The most critical of these is the decline in soil fertility and vegetation cover caused by increased drought, which affects community composition and ecosystem function [3]. Understanding how drylands respond to continuous environmental changes is particularly important for sustainable development.

Variations in climate, vegetation cover, and land use are major drivers of global change [4], and desert ecosystems exhibit a heightened sensitivity to alterations in climate and vegetation cover. Given that water is the primary limiting factor for biological activity in drylands and seasonal and interannual changes in precipitation have profound impacts on the community composition and ecological function of arid ecosystems [5], the importance of climate as a driver of dryland structure and function has been widely accepted. Equally important, soil nutrient cycling and the stoichiometric ratio of plant leaf nutrients are key indicators that determine community structure and function and impact ecosystem stability [6]. However, under the influence of the macroclimate, it is still unclear how the soil–plant nutrition relationship regulates ecosystem stability in desert ecosystems. 

Soil and plant nutrients are affected by climate factors. Climate change affects species composition, microbial environment, and litter decomposition, thereby affecting soil nutrient cycling and supply [7]. The soil C:N:P in different climatic zones of China has spatial heterogeneity, which is mainly affected by temperature and precipitation [8]. For example, an increase in the aridity index will reduce soil C and N, plant abundance, and microbial activity and produce more inorganic phosphorus, leading to soil nutrients’ stoichiometry imbalance in drylands [9]. An increase in the aridity index may also have a negative impact on the availability of micronutrients by increasing the soil pH while reducing the soil organic matter. Studies on leaf nutrients in a global dataset revealed that leaf nitrogen and phosphorus exhibited a negative correlation with the mean annual temperature (MAT) and the mean annual precipitation (MAP), and the leaf nitrogen–phosphorus ratio showed an opposite trend [10,11]. However, He et al. [12] revealed that there was no strong correlation observed between the MAT, the MAP, and leaf nutrients. Therefore, under conditions of extreme drought, the clarification of the variation patterns in soil and plant nutrients across the gradient of key climate factors in desert ecosystems remains a necessity.

The nutrient content in plant leaves exhibits a strong relationship with the nutrient composition of the soil in which they grow [13], and plant productivity is positively correlated with soil nutrient concentrations [14]. The influence of temperature and precipitation on soil microbial activity as well as its physical and chemical properties is significant, in turn creating different conditions for plant growth. Similarly, under different local conditions, such as soil water content, salt content, light intensity, and litter layer, there are different soil–plant nutrient cycling mechanisms, and these variations lead to significant differences in the nutrient composition of plant leaves [15,16]. The soil nutrients available to plants have a significant impact on plant growth and ecosystem productivity. Nitrogen (N) and phosphorus (P) are often the primary nutrients that restrict plant growth, while the strategies employed by plants to utilize these nutrients hinge on the different stages of soil development [17]. No matter what the soil age is, nitrogen and phosphorus have a greater impact on leaf nutrient concentrations. The overall fertility effects of soil N and P jointly explain the great variation in leaf N and P. With the succession of ecosystems on a time scale, N and P change species composition and structure and affect the net primary productivity of ecosystems [18,19]. The relationship between major nutrients in soil and plants in desert ecosystems needs further study.

Huang et al. [20] defined the time stability of an ecosystem as the ratio of the average NDVI over the years (NDVI mean) to the average NDVI standard deviation (NDVI SD) over the same period, revealing that the time stability of an ecosystem can be improved by increasing the mean NDVI value or reducing the standard deviation of the NDVI to above the mean value. Li et al. [21] revealed that soil nutrient resources are closely linked to plant diversity. Soil organic carbon exerts a positive influence on plant growth. Plant diversity enhances soil microbial activity and improves soil biodiversity and its ability to absorb nutrients from plant litter, thereby improving the stability of the ecosystem [22]. In addition, in the climate context, nitrogen and phosphorus, as the main limiting nutrients of terrestrial ecosystems, directly affect plant productivity and ecosystem stability [23,24]. The soil–plant nutrition relationship is an important regulatory mechanism for ecosystem stability. Therefore, it is particularly important to explore the relationship between soil and plant nutrients and the mechanism of their effects on ecosystem stability under drought stress.

At present, research on the factors affecting ecosystem stability mainly focuses on climate warming, species diversity, and microbial regulation [25,26], but there are few studies on the mechanism of the effect of soil nutrient transport on the temporal stability of plant productivity under drought stress. Studies have shown that the temperature in the Qaidam Basin has shown a significant upward trend in the past 60 years and that the warming rate is significantly higher than that in other areas of the Qinghai–Tibet Plateau [27,28], allowing the Qaidam Basin to better reflect the response mechanism of desert ecosystems to continuous environmental changes. Therefore, the objectives of this study were the following: (1) explore the varying patterns in soil and plant nutrients across the drought gradient within the desert vegetation zone in the Qaidam Basin; (2) analyze whether soil nutrients (N, P, K, C, AP, and AHN) have specific effects on plant leaf nutrients; and (3) determine the driving effect and influence path of soil, plant nutrients, and environmental factors on ecosystem stability.

## 2. Materials and Methods

### 2.1. Study Area

**Figure 1:**
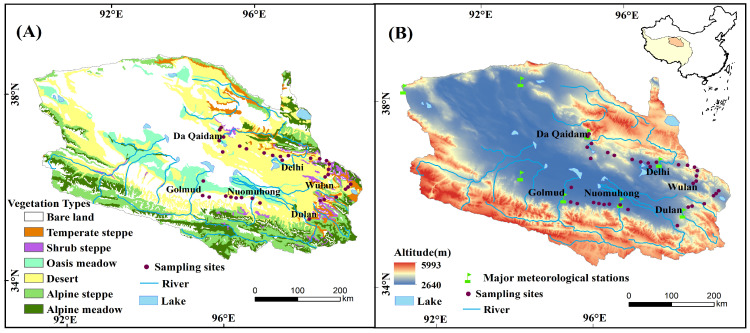
Vegetation (**A**), altitude (**B**), location of major meteorological stations and sampling sites in the Qaidam Basin.

The Qaidam Basin is situated in the northeastern part of the Qinghai–Tibet Plateau in China, which is the world’s highest basin with an altitude ranging from 5993 m to 2640 m (Figure 1). The MAP is generally less than 200 mm, the MAT is generally less than 5℃, and the annual potential evapotranspiration is more than 2000 mm, which has the characteristics of high cold, high drought and high salinization.

The Qaidam Basin is dominated by a desert ecosystem and its ecological environment is fragile. Soil types are mostly desert soils with low nutrient content, and individual sample soils are inland salt soils or salt crusts. Desert vegetation is the main vegetation cover type in the Qaidam Basin, occupying about 50% of the vegetation cover area. The plant life forms include shrubs, semi-shrubs and perennial herbs, which have high resistance to cold, drought and saline-alkali. The dominant species are *Ephedra sinica*, *Sympegma regelii*, *Ceratoides latens*, *Haloxylon ammodendron*, *Kalidium foliatum*, *Tamarix chinensis Lour*, *Salsola abrotanoides*, *Achnatherum splendens*, etc., whose growth status reflects the nutrient utilization of vegetation and its relationship with soil nutrient elements and the adaptation characteristics of arid environments in high altitude desert areas.

### 2.2. Field Survey and Sampling 

Desert vegetation is the main vegetation type in the Qaidam Basin (Figure S1), accounting for 50% of the vegetation cover, which is in the horizontal zone at the bottom of the basin, and is a product and representative of the desert climate. We set 35 sampling sites along the precipitation gradient in the typical desert vegetation distribution area of the Qaidam Basin during the peak growth period for plants from July to August 2016, involving 9 shrub communities and 1 perennial herb community type in desert vegetation, which are representative. Plant quadrats with different sizes of 2 m × 2 m, 5 m × 5 m and 10 m × 10 m were set according to the plant life form. In general, 3–5 quadrats were set in each sample plot to collect all plant species in the community. In total, 3–5 samples of each plant were collected, mainly plant leaves, and the leaves of the same plant species were mixed to collect 100 plant samples. Plant coverage, plant height, species and altitude were recorded during sampling. Three parallel soil profiles were randomly investigated in each plant sample site. The ground litter should be removed first during sampling. Three soil samples with different depths of 0–10 cm, 10–30 cm and 30–50 cm were selected by the mechanical stratification method, and about 500 g soil samples were selected by the quartering method. Fresh soil samples were packed into numbered self-sealing bags and brought back to the laboratory for air drying. Since no soil samples were collected at 15 sampling sites at 30–50 cm (some deep soils in desert areas are composed of large gravel), a total of 90 soil samples were collected.

### 2.3. Experimental Methods and Index Calculation

Plant samples were washed and air-dried to remove surface moisture. The samples were dried in an oven until they were permanently dry and were ground and sifted through a 0.15 mm sieve and put into plastic bags for the determination of plant nutrient elements. After removing the visible animal and plant remains and gravel, the soil was air-dried in a cool and ventilated place. The air-dried soil was mixed evenly. The appropriate amount of soil was selected through the quartering method with 0.15 mm, 0.25 mm and 2 mm sieves for the determination of soil nutrient elements. 

The determination of nutrient elements in plant samples: the organic phosphorus in the samples was converted into inorganic phosphate by H_2_SO_4_ and H_2_O_2_. At the same time, the organic nitrogen was also converted into inorganic ammonium salt. Leaf total phosphorus (LTP, g/kg), and total potassium (LTK, g/kg) were measured by the test solution. Leaf total carbon (LTC, g/kg) and total nitrogen (LTN, g/kg) were quantified by a Euro Vector EA3000 element analyzer from Italy.

Soil total phosphorus (STP, g/kg), soil total potassium (STK, g/kg), soil available phosphorus (AP, mg/kg), and alkali-hydrolyzed nitrogen (AHN, mg/kg) were measured, respectively, by the alkali fusion–molybdenum antimony resistance colorimetric method, alkali fusion–flame photometric method, sodium bicarbonate extraction–anti-color comparison method, and alkaline dissolution diffusion method, soil total nitrogen (STN, g/kg) and organic carbon (SOC, g/kg) content were determined using an Italian Euro Vector EA3000 element analyzer, and 1 mol/L of hydrochloric acid was added for pretreatment before measurement. The air-dried soil and deionized water were mixed by 1:5 to form a suspension, and then the suspension was extracted to measure the soil pH value. The conductivity value was measured by the conductivity meter, and the conductivity value served as an indicator for assessing the soluble whole salt content within the soil (SS, ms/cm). A certain amount of fresh soil was weighed by a thousandth of a balance, its dry weight was measured after drying for 48 h at 105 °C, and its water content was calculated (SWC, %). 

In our study area, we calculated the community-weighted mean value (CWM) for each sample plant’s leaf nutrient content, utilizing the following formula [29]:

(1) $$CWM_{j}=\sum_{i=1}^{n}P_{ij}T_{ij}$$

Specifically, P_ij_ represents the relative cover of species i in sampling site j, while T_ij_ denotes the mean of the trait values of species i within that same sampling site. CWMj is the community-weighted mean value of traits of each species in sampling site j.

### 2.4. Climate Data Collection and Processing

The temperature and precipitation data were retrieved from the National Meteorological Data Science Center (http://data.cma.cn/site/index.html, accessed on 1 September 2023). According to the meteorological data of 19 sites around the Qaidam Basin, the KRIDGING interpolation method in ArcGIS was used to obtain the temperature and precipitation data. Potential evapotranspiration data (PET) were obtained from the 1 km resolution “Potential evapotranspiration 1901–2020 in the Qaidam Basin” provided by the Chengdu Institute of Mountain Sciences, Chinese Academy of Sciences. The MAT, MAP and PET of sampling sites from 1961 to 2020 were extracted according to geographical coordinates, and the aridity index (AI) was calculated by the ratio of PET to MAP [30].

### 2.5. Climate Data Collection and Processing

Normalized Difference Vegetation Index (NDVI) is closely related to vegetation productivity and can replace the value of productivity, which has high monitoring accuracy for grassland and sparse vegetation [31]. In this paper, NDVI data from 2000 to 2020 were downloaded using the Google Earth Engine (https://ladsweb.modaps.eosdis.nasa.gov/, accessed on 1 October 2023) and the maximum value composited method obtained the maximum NDVI in every month and every year. The NDVI mean and standard deviation of 35 sampling sites in the Qaidam Basin were extracted in ArcGIS 10.4. Zhang et al. [32] defined the ecosystem stability as S = μ/σ, where μ and σ are the annual mean and standard deviation of the net primary productivity of the ecosystem. Based on this premise, the current study employs NDVI as a surrogate measure of vegetation productivity, and the calculation formula for ecosystem stability is defined as:

(2) $$S=\frac{{NDVI}_{Mean}}{{NDVI}_{SD}}$$

### 2.6. Data Analysis

First, the aridity index (AI) of sampling sites was divided according to the ArcGIS 10.4 natural breakpoint method. If the aridity index was less than 10, it was mild drought, 10–20 was moderate drought, and greater than 20 was severe drought (Table 1).

Secondly, the Kruskal–Wallis test (K-W test) was utilized to assess and contrast the variations in soil properties, plant nutrients, and climate factors along drought gradient.

In order to explore whether climate factors have an impact on soil and plant nutrients, as well as the change rule of soil and plant nutrients with drought gradient, this study analyzed soil nutrient elements (STN, STP, STK, SOC, AP, AHN), plant nutrient elements (LTC, LTN, LTP, LTK), the quantitative ratios of soil and plant nutrients (C:P, C:N, N:P), which were taken as response variables, and climate factors (MAT, MAP, AI), which were taken as explanatory variables. Bivariate regression analysis was used to test the association between response variables and explanatory variables. In addition, logarithmic transformation of soil and leaf nutrient data was carried out to meet the normal distribution, and regression analysis was utilized to examine the relationship between plant and soil nutrient elements.

Next, in order to explore the variation trend in NDVI in the Qaidam Basin on a spatio-temporal scale, this study used Theil–Sen Median and Mann–Kendall methods to analyze the variation trend in NDVI in the Qaidam Basin from 2000 to 2020 and obtained the percentage of different change trends in the area. A multivariate linear model was used to assess the percentage of variance explained by soil, plant nutrients, and environmental factors on ecosystem stability, and to determine the covariance among explanatory variables and screen the optimal model based on the variance inflation factor (VIF).

Finally, utilizing structural equation modeling (SEM), we delved into the most important influence soil nutrients exert on plant nutrients, as well as the intricate pathway connecting the impact of soil, plant nutrients, and environmental factors on the stability of ecosystems. Additionally, a stepwise regression analysis was conducted to refine the model by sequentially eliminating variables of least significance, ultimately yielding an optimal model that best captures the underlying relationships.

All statistical analysis and mapping were performed using R 4.3.2, and maps were generated by ArcGIS10.4.

## 3. Results

### 3.1. Variation Characteristics of Soil and Plant Nutrient Elements along Drought Gradient

**Figure 2:**
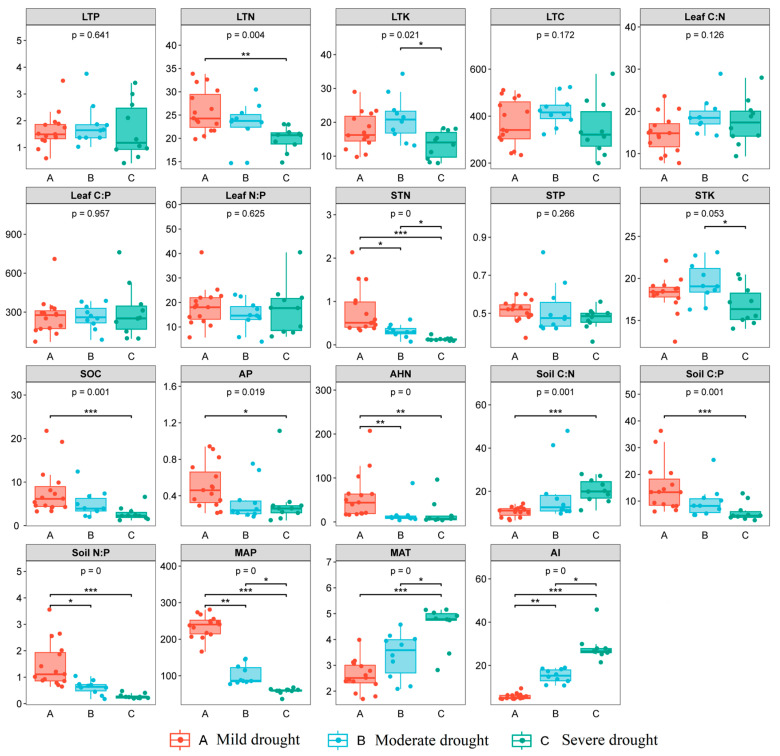
Differences in soil, plant nutrients and climate factors in different drought gradients. Note: Plant leaf nutrients included the community weighted means (CWMs) of total carbon (LTC), total nitrogen (LTN), total phosphorus (LTP), total potassium (LTK) and stoichiometric ratios of carbon, nitrogen, and phosphorus (Leaf C:N, Leaf C:P, Leaf N:P) of all plants at each sampling sites. Soil nutrients included organic carbon (SOC), total nitrogen (STN), total phosphorus (STP), total potassium (STK), available phosphorus (AP), alkali-hydrolyzed nitrogen (AHN), and soil stoichiometric ratios of carbon, nitrogen, and phosphorus (Soil C:N, Soil C:P, Soil N:P) at the depth of 0-50 cm in the topsoil. Climate factors include mean annual precipitation (MAP), mean annual temperature (MAT) and annual drought index (AI). The difference significant at 0.001, 0.01 and 0.05 levels were indicated by ***, **, and *, respectively.

There were significant differences in LTN between mild and severe droughts (*p* < 0.01), and LTK between moderate and severe droughts (*p* < 0.05), while other leaf nutrient concentrations did not differ significantly under different drought gradients (*p* > 0.05). Except for STP, all other soil nutrients showed differences under different drought gradients (*p* < 0.05), among which SOC, AP, soil C:N, and soil C:P showed significant differences between mild and severe droughts, while STK showed significant differences between moderate drought and severe drought, and other soil nutrient contents (STN, AHN, soil N:P) showed significant difference between mild, moderate, and severe droughts (*p* < 0.05, Figure 2). It is worth noting that the contents of STN, STP, SOC, AP and AHN in mild drought areas are significantly higher than those in moderate and severe drought areas.

There was a significant negative correlation between MAT and various soil nutrients including STN, STP, STK, SOC, AHN, soil C:P, and soil N:P (*p* < 0.05), however, AP decreases and then increases with increasing MAT (*p* < 0.01), showing a nonlinear trend. STN, SOC, soil C:P, and soil N:P showed a significant positive correlation with MAP (*p* < 0.01), while AP and AHN showed a nonlinear trend in decreasing and then increasing with MAP(*p* < 0.01). Similarly, STN, AP, SOC, AHN, C:P, and N:P decreased first with AI and then increased to the maximum point of AI (*p* < 0.01). Soil C:N has a positive correlation with MAT and AI and an opposite trend with MAP (Figure S2; *p* < 0.05). Soil C:N reflects the conversion capacity of soil organic matter and is used to measure soil quality. The soil C:N ratio serves as an indicator of the soil’s ability to convert organic matter, thus serving as a measure of soil quality. As the C:N ratio increases, the decomposition of soil organic matter becomes less efficient, leading to a deterioration in soil fertility conditions. Our findings reveal that as AI increases, the overall fertility of the soil decreases.

At the community scale, LTN exhibited a positive correlation with MAP, while it showed a significant decrease with the rising AI (Figure S3; *p* < 0.01). However, there was no significant relationship between other leaf nutrients and MAT, MAP, and AI (Figure S3; *p* > 0.05).

### 3.2. Soil–Plant Nutrient Element Relationship of Desert Vegetation

**Figure 3:**
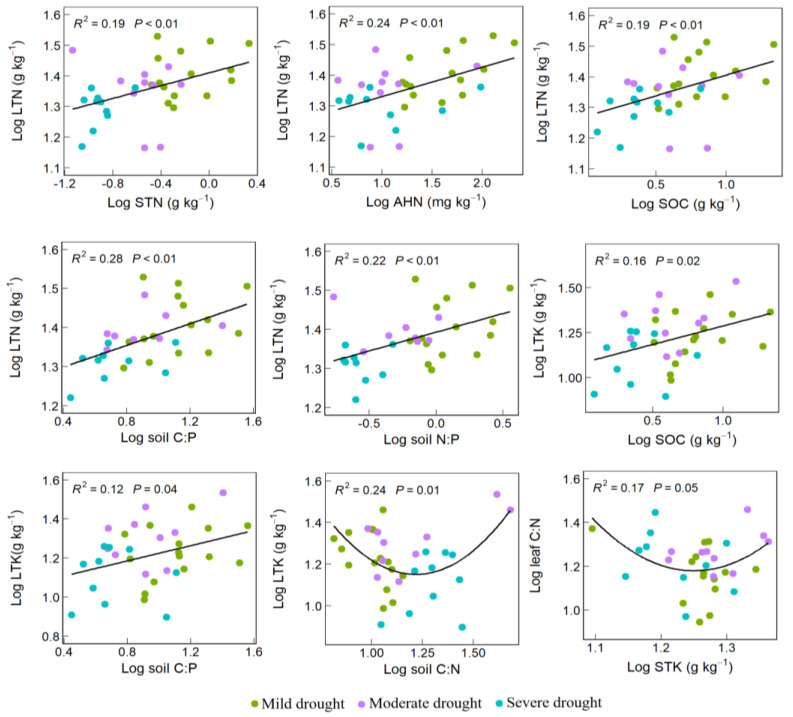
Relationship between soil and plant nutrients. Note: Soil total nitrogen (STN), alkali-hydrolyzed nitrogen (AHN), organic carbon (SOC), soil total potassium (STK), soil stoichiometric ratios of carbon, nitrogen, and phosphorus (soil C:N, soil C:P, soil N:P). Leaf total nitrogen (LTN), total potassium (LTK), leaf stoichiometric ratios of carbon and nitrogen (leaf C:N).

At the community scale, LTN exhibited a positive correlation with STN, AHN, SOC, soil C:P, and soil N:P (*p* < 0.01). LTK displayed a significant positive correlation with SOC and soil C:P, and decreased first and then increased with soil C:N increasing. Leaf C:N showed a nonlinear trend in first decreasing and then increasing with STK increasing (Figure 3; *p* < 0.05).

The relationship between leaf nutrients, soil nutrients and environmental factors was further explored through redundancy analysis (RDA). We found that: the total variation in leaf nutrient traits was attributed to 23.1% by environmental variables and soil nutrients. The cumulative explanation of the initial two axes constituted 84.4%, with the first axis being the primary determinant (Figure S4). Hierarchical partitioning (HP) was performed on STN, SOC, AP, AHN, SWC, SS and MAT to analyze the contribution degree and importance of soil and environmental factors and obtain the important ranking of each factor. Among them, SOC (28.05%), AHN (19.44%), SS (16.28%), MAT (12.94%) and SWC (10.35%) had high interpretation rates, indicating that SOC, AHN, SS, MAT and SWC were important factors affecting the leaf nutrient content of plant communities.

### 3.3. Productivity Change Trend and Ecosystem Stability in the Qaidam Basin

**Figure 4:**
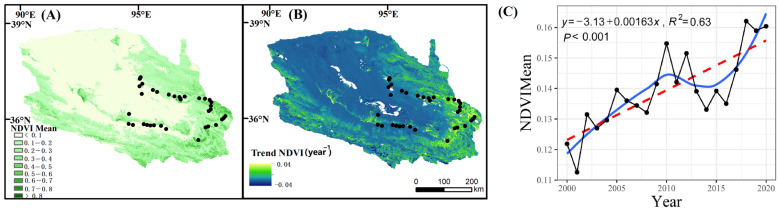
Mean value (**A**) and change trend (**B**,**C**) of NDVI in the Qaidam Basin from 2000 to 2020. Trend NDVI (year^−1^): Annual change trend of Normalized Difference Vegetation Index. The black broken line scatter is the annual NDVI mean, the red dashed line is the unary linear regression fitting result, and the blue solid line is the smooth fitting result of the locally weighted scatter plot.

On the whole, the mean NDVI value of the Qaidam Basin showed a semi-annular, diminishing gradually from the southeast to the northwest, and the mean NDVI value of the multi-year growing season was 0.14. There was an overall improving trend in NDVI within the basin from 2000 to 2020, increasing at a rate of 0.015 per decade. The areas that exhibited a mean NDVI value below 0.1 were primarily concentrated in the desert regions located to the south of Da Qaidam–Delhi and to the west of Xiangride–Dulan. The regions with higher mean NDVI values are mainly located in the temperate grassland and alpine grassland, alpine meadow area in the east of Xiangride–Dulan, oasis meadow area of Golmud–Nuomuhong in the middle of the basin, alpine grassland and alpine meadow area in the south of the basin (Figure 4).

The time series analysis of the mean NDVI value in the Qaidam Basin from 2000 to 2020 showed that the mean NDVI value fluctuated and increased with time (Figure 4, *p* < 0.001). Among them, the stable area accounted for 33.23%, the area with an obvious improvement of vegetation cover accounted for 50.24% of the total area of the basin, while the area with vegetation degradation accounted for only 2.02% (Figure S5, Table S1). 

**Figure 5:**
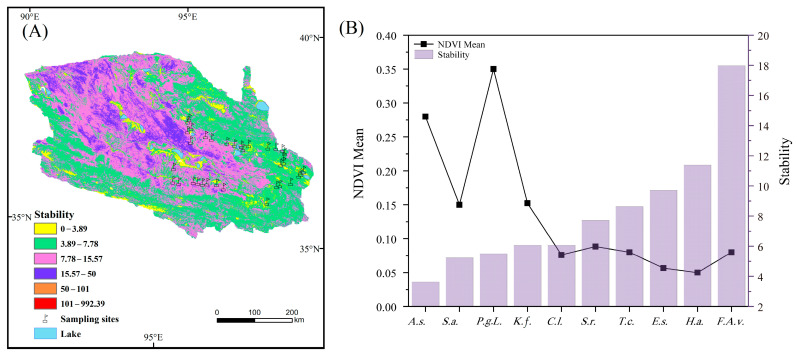
Ecosystem stability (**A**), mean NDVI values and ecosystem stability characteristics of different desert plant communities (**B**) in the Qaidam Basin. Note: *A.s.*: *Achnatherum splendens*; *S.a.*: *Salsola abrotanoides*; *P.g.L.*: *Potentilla glabra Lodd*; *K.f.*: *Kalidium foliatum*; *C.l.*: *Ceratoides latens*; *S.r.*: *Sympegma regelii*; *T.c.*: *Tamarix chinensis Lour*; *E.S.*: *Ephedra sinica*; *H.a.*: *Haloxylon ammodendron*; *F.A.v.*: *Folium Apocyni Veneti*.

NDVI stable and unchanging bare land in the interior of the basin, alpine meadow, alpine steppe, and temperate steppe ecosystems on the basin margins are more stable, and oasis meadow and desert are less stable. In the desert vegetation area we focused on, the NDVI of 35 sampling sites ranged from 0.04 to 0.36, with an average value of 0.13, and the changing trend in NDVI ranged from stable to significantly improved. The community types with higher NDVI were *Achnatherum splendens* and *Potentilla glabra Lodd*, while those with lower NDVI were *Ephedra sinica* and *Haloxylon ammodendron*. Ecosystem stability ranges from 2.92 to 17.97, with an average of 6.98, indicating low stability. The community with the highest ecosystem stability was Folium Apocyni Veneti and the lowest was Achnatherum splendens (Figure 5).

### 3.4. Effects of Soil Nutrients, Plant Nutrients, and Environmental Factors on Ecosystem Stability

The ecosystem stability of the 10 community types involved in sampling sites was mainly affected by LTC, STN, MAT and AP (*p* < 0.05). LTC could explain 15% of the variance of ecosystem stability, STN could explain 18.92%, MAT could explain 7.48% and AP could explain 7.54%. Collectively, these four factors accounted for 48.94% of the variance in stability (Table 2).

## 4. Discussion

### 4.1. Effects of Arid Environment on Soil and Plant Nutrients

There is abundant evidence that climate change-induced heat and drought have led to increased tree mortality, with significant impacts on vegetation productivity [33]. Variations in temperature and precipitation affect soil parent material weathering rates, soil nutrient supply, and plant growth [34]. In terms of plant nutrients, the integration of these processes controls the accumulation and removal rates of C, N, and P in leaves [10]. The Temperature–Plant Physiology Hypothesis (TPPH) postulates that in colder environments, plants enhance their leaf N and P as a compensatory mechanism to mitigate the reduced physiological efficiency resulting from low temperatures. Conversely, as temperatures rise, the concentrations of leaf N and P diminish. In essence, N and P levels compensate for changes in temperature, which in turn regulates the rate of C acquisition [35]. In terms of soil nutrients, water affects soil nutrient content by directly affecting soil microbial decomposition rates [36], and by affecting plant growth and limiting the amount of litterfall [37].

As the severity of drought intensifies, the soil organic matter from plants and microorganisms decreases, the SOC and STN content decreases, and the vegetation changes from grassland and savanna to shrubland to better adapt to the poor nutrition sandy soil [3]. The primary source of phosphorus content in soil originates from the mechanical weathering process of rocks. The increase in drought degree reduced the plant abundance, intensified the weathering of a large number of bare rocks, and increased the P concentration [38]. In our research, except for STP, other soil nutrients showed differences under different drought degrees. The soil nutrient levels exhibited a negative correlation with MAT and a positive correlation with MAP and showed a general trend of first decreasing and then increasing with AI. STN, AP, SOC, and AHN showed nonlinear relationships with AI. The nutrients increased at the 35th sampling site (severe drought). The possible reason was that the SWC and SS at the 35th sampling site were high, resulting in higher soil nutrient content than other sites with severe drought. In desert ecosystems, we also need to pay attention to differences in local environmental factors such as SWC, SS, etc.

Globally, Reich and Oleksyn [10] found that LTP and LTN decreased slightly with the increase in MAT, whereas leaf N:P increased with the increase in MAT. In this study, on the community scale of typical desert vegetation in the Qaidam Basin, the relationships of leaf nutrients with MAT, MAP, and AI were not significant, except for leaf nitrogen content, which was significantly correlated with MAP and AI. This may be because the environment in the desert area is relatively complex. Under the background of drought, the regularity of large-scale climate factors may be covered by the influence of small-scale local factors. The strong change trends in leaf nutrients were not detected in the sampling sites along the gradient of MAT (ranging from 1.69 °C to 5.15 °C) and MAP (ranging from 36.02 mm to 280.46 mm) in the study area. Nonetheless, there was no significant correlation observed between either MAT or MAP and leaf nutrient levels, which was consistent with previous research as well [12]. Climate indirectly affects nutrient concentrations in plant organs by influencing soil properties and vegetation composition [39]. In addition, the impact of climate factors on plant nutrient elements may have different forms in different biological taxa and species composition [11,40].

### 4.2. Soil–Plant Nutrient Element Relationship

Plants often prioritize allocating nutrients to their leaves initially, aiming to guarantee robust growth and vitality, absorbing nutrients from fresh leaves is a strategy for nutrient circulation and storage in plants [41]. When nutrients are limited, trees use nutrients stored in woody stems to meet leaf requirements, and nutrient stoichiometry of fresh leaves allows assessment of tree nutrient utilization strategies [42]. The variation of leaf nutrient concentration along the soil fertility gradient has been confirmed [43,44,45].

Previous research has indicated that soil nutrients serve as the primary driving factors influencing the composition and dynamics of plant communities within arid desert ecosystems [13,46]. In the Qaidam Basin, we found that leaf nitrogen content was significantly positively correlated with STN, AHN, and SOC, and leaf potassium content increased with SOC increasing. This finding basically corresponds with Zhang et al. [47], who found that STN and STP content was significantly positively correlated with LTN and LTP concentrations, while leaf C:P, N:P had no strong correlation with soil nutrients [48]. This is because the effective phosphorus available to plants comes mainly from rock weathering and is weakly related to soil nutrients [49]. In arid regions, high soil pH and low micronutrient availability, combined with limiting moisture conditions, in turn affect leaf carbon [50]. By comparing the C:N:P of soil and plant leaves in the Qaidam Basin and the whole country, it was found that SOC in the study area was low, and showed characteristics of N limitation and P enrichment. The growth of plants in the study area was constrained by the availability of nitrogen (N) and phosphorus (P), and was more prone to N limitation [51]. The heightened nitrogen limitation observed in halophytes within arid desert ecosystems aligns with the findings of Wang et al. [52]. Consequently, SOC and STN in this study emerged as the key factors influencing the nutrient composition of plant leaves.

Plant leaf nutrients are also affected by other soil nutrient elements, environmental conditions, and species types [9,53,54]. Our redundancy analysis of soil and environmental factors and plant nutrients indicated that SS exhibited a notable positive correlation with LTN while displaying a negative correlation with LTC. Additionally, the MAT was inversely correlated with the overall nutrient content of plant leaves. The elevated leaf nitrogen concentration observed in drought- and salt-tolerant species is potentially attributed to the greater accumulation of non-protein nitrogen in halophytes as a response to salt stress conditions [55,56]. In addition, salt stress leads to reduced stomatal conductance and osmoregulation [57], inhibition of photosynthesis in plants, and reduced rates of C fixation [52], further affecting plant growth and development. In global-scale studies of leaf nutrients, the decrease in leaf nutrients with increasing MAT has been confirmed [40].

### 4.3. Influence Pathway of Soil, Plant Nutrients and Climate Factors on Ecosystem Stability

Previous studies have shown that desert shrub ecosystems are less stable than grassland and forest ecosystems, and evergreen broadleaf forest ecosystems are more stable and more resistant to drought than other biological communities [58]. This is because the drought resistance of vegetation productivity increases with precipitation, forest ecosystems are highly resistant, and ecosystems are more stable over time [59], while grasslands and shrublands are more vulnerable to drought stress, resulting in low drought resistance and decreased species richness, thus affecting ecosystem stability [60]. Similarly, the ecosystem stability of the desert vegetation distribution area in the Qaidam Basin is low.

**Figure 6:**
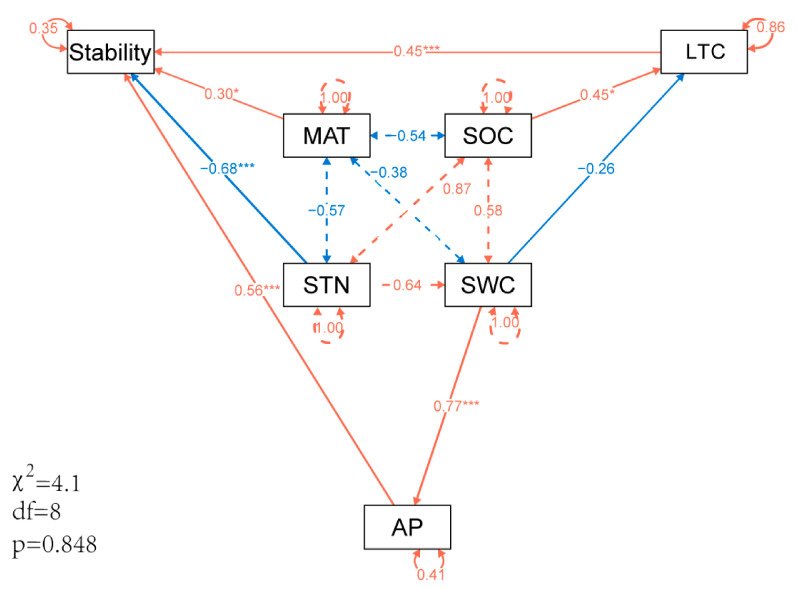
Direct and indirect effects of soil, leaf nutrients and environmental factors on ecosystem stability in structural equation model. Note: Leaf total carbon (LTC), soil organic carbon (SOC), total nitrogen (STN), available phosphorus (AP), mean annual temperature (MAT), soil water content (SWC). The red and blue lines represent positive and negative effects, respectively, with significant differences at 0.001 and 0.05 levels indicated by *** and *, respectively.

Chen et al. [61] revealed that a higher photosynthetic rate and high LTC can mitigate high-temperature stress on plants, and desert shrubs are less sensitive to temperature and precipitation with increasing LTC. Equally important, plants adopt higher leaf carbon-nitrogen ratios as drought tolerance strategies in a water-limited environment [62]. Our results confirmed that LTC has a forward and positive effect on ecosystem stability. SOC indirectly affects ecosystem stability by having a significant positive effect on LTC (*p* < 0.05), SWC has a direct negative effect on LTC, and SOC and SWC can explain 14% of LTC variance. Furthermore, our research revealed that STN had a significant negative effect on ecosystem stability, which may be that nitrogen enrichment increased the synchrony between dominant species, thereby significantly increasing the synchrony of the entire community and reducing plant species diversity. Concurrently, alterations in the composition of dominant species brought about by nitrogen input have significantly augmented population variability. This surge in species synchrony and population variability has subsequently diminished the dominance and stability of the dominant species, ultimately resulting in a decrement in the overall ecosystem stability [63,64]. Plant growth in alpine meadows was not inhibited but restricted by soil available nitrogen, and nitrogen enrichment, on the other hand, limited plant growth [24]. On the contrary, phosphorus enrichment can even increase the number of species in nutrient-poor or high-elevation areas, improving species richness and stability of dominant species, thus contributing to productivity stability [24]. Our results also confirmed that AP had a significant positive effect on ecosystem stability. SWC had a significant positive effect on AP (*p* < 0.001), which could explain 59% of the variance of AP (Figure 6). 

Plant productivity in alpine ecosystems is closely related to water conditions, plant drought tolerance is significantly correlated with SWC, and ecosystem stability increases with increasing SWC [65]. In this study, SWC has a positive and effective indirect effect on ecosystem stability by positively affecting AP. Regarding the impact of temperature on ecosystem stability, most studies believe that higher precipitation and lower temperature have a positive impact on stability [66,67]. However, some studies have found that warming increases NPP and biodiversity, which is positive for the time stability of productivity [68]. We observed that MAT has a positive impact on ecosystem stability. This may be attributed to the fact that the study area falls within an alpine desert ecosystem, where warmer temperatures tend to extend the growth season for plants. Consequently, MAT is positively correlated with plant productivity, ultimately enhancing the stability of the ecosystem [69,70]. In general, LTC, MAT, and AP exert a strong positive impact on stability (*p* < 0.05), and STN has a strong negative effect on stability (*p* < 0.001). These four factors can explain 65% of the variance of stability (Figure 6).

## 5. Conclusions 

In summary, the results of this study show that soil nutrients have obvious regularity of distribution along the drought gradient, and the soil nutrient content is high in areas with low temperatures and high precipitation. Soil alkali-hydrolyzed nitrogen and organic carbon had higher interpretation rates of leaf nutrients and were significantly positively correlated with leaf nitrogen and potassium content. In general, the soil nutrient content in mild drought areas was significantly higher than that in severe drought areas, and soil carbon and nitrogen were the key factors affecting leaf nutrients. By analyzing the temporal stability of vegetation productivity and its influencing factors in the Qaidam Basin, it is concluded that the vegetation change in the Qaidam Basin from 2000 to 2020 shows an improving trend, but the ecosystem stability in the desert vegetation distribution area is low. Soil-available phosphorus has a positive effect on ecosystem stability, while nitrogen has a negative effect. In addition, leaf carbon and mean annual temperature had significant positive effects on stability. Our results emphasize the mechanism of soil action on ecosystem stability directly or indirectly through plant nutrients. This study focuses on the effects of soil and plant nutrients on ecosystem stability under drought stress, and it is suggested that more work should be conducted in the future to study the long-term effects of trade-offs or synergies of water, carbon and nutrient utilization on plant productivity and ecosystem stability.

## Figures and Tables

**Table 1:** Geographic location, altitude (Alt), soil type, community type, mean annual temperature (MAT), mean annual precipitation (MAP), aridity index (AI), and drought gradient of sampling site.

Site	Lat. (°N)	Long. (°E)	Alt. (m)	Soil Type	Community Type	MAP (mm)	MAT (°C)	AI	Drought Gradient
1	36.35	95.09	2889	Brown calcic soil	*Sympegma regelii*	58.49	5.15	27.80	severe drought
2	36.33	95.27	2968	Grey brown desert soil	*Ephedra sinica*	62.87	5.03	25.14	severe drought
3	36.38	95.70	2847	Grey brown desert soil	*Ephedra sinica*	60.8	4.8	26.49	severe drought
4	36.37	95.85	2890	Grey brown desert soil	*Sympegma regelii*	61.33	4.75	25.83	severe drought
5	36.36	95.98	2824	Grey brown desert soil	*Tamarix chinensis Lour*	61.01	4.74	26.28	severe drought
6	36.38	96.13	2765	Grey brown desert soil	*Tamarix chinensis Lour*	57.78	4.77	27.80	severe drought
7	36.38	96.41	2853	Grey brown desert soil	*Ceratoides latens*	53.31	4.91	29.82	severe drought
8	36.29	96.63	2847	Grey brown desert soil	*Haloxylon ammodendron*	85.12	4.57	18.48	moderate drought
9	36.03	97.97	3160	Brown calcic soil	*Achnatherum splendens*	203.83	3.11	7.09	mild drought
10	36.44	98.23	3255	Brown calcic soil	*Achnatherum splendens*	206.2	3.17	6.40	mild drought
11	36.47	98.33	3320	Brown calcic soil	*Kalidium foliatum*	216.75	2.96	5.78	mild drought
12	36.54	98.65	3530	Chestnut soil	*Kalidium foliatum*	244.51	2.38	4.47	mild drought
13	36.72	98.88	3173	Chestnut soil	*Kalidium foliatum*	267.19	1.9	4.96	mild drought
14	36.77	98.95	3069	Chestnut soil	*Kalidium foliatum*	273.07	1.79	4.78	mild drought
15	36.84	99.01	3125	Chestnut soil	*Achnatherum splendens*	280.46	1.69	4.72	mild drought
16	36.96	98.31	2945	Chestnut soil	*Sympegma regelii*	231.44	2.78	6.20	mild drought
17	37.02	98.39	3132	Chestnut soil	*Kalidium foliatum*	240.11	2.61	5.69	mild drought
18	37.13	98.39	3276	Chestnut soil	*Kalidium foliatum*	248.91	2.43	4.70	mild drought
19	37.24	98.40	3379	Chestnut soil	*Achnatherum splendens*	255.04	2.27	4.43	mild drought
20	37.32	98.32	3503	Chestnut soil	*Achnatherum splendens*	245.76	2.37	4.52	mild drought
21	37.35	98.13	3466	Chestnut soil	*Potentilla glabra Lodd*	237.33	2.51	4.61	mild drought
22	37.34	97.89	3204	Chestnut soil	*Salsola abrotanoides*	212.62	3.05	6.12	mild drought
23	37.37	97.28	2971	Grey brown desert soil	*Kalidium foliatum*	165.65	3.98	9.33	mild drought
24	37.26	97.13	2862	Grey brown desert soil	*Ceratoides latens*	145.96	4.14	10.65	moderate drought
25	37.33	97.07	2905	Grey brown desert soil	*Kalidium foliatum*	144.17	4.01	10.80	moderate drought
26	37.35	96.86	2829	Inland salt soil	*Kalidium foliatum*	124.76	3.94	12.71	moderate drought
27	37.38	96.62	2982	Grey brown desert soil	*Sympegma regelii*	114.6	3.79	13.57	moderate drought
28	37.44	96.13	3621	Grey brown desert soil	*Salsola abrotanoides*	87.46	3.38	14.73	moderate drought
29	37.49	95.95	3336	Grey brown desert soil	*Sympegma regelii*	82.94	3.14	17.28	moderate drought
30	37.65	95.49	3248	Grey brown desert soil	*Ceratoides latens*	76.76	2.56	18.87	moderate drought
31	37.86	95.39	3308	Chestnut soil	*Sympegma regelii*	85.11	2.08	15.80	moderate drought
32	37.80	95.36	3188	Grey brown desert soil	*Ceratoides latens*	80.93	2.18	18.20	moderate drought
33	37.56	95.38	3184	Inland salt soil	*Ceratoides latens*	67.39	2.81	21.38	severe drought
34	37.33	95.51	3035	Salt crust	*Ephedra sinica*	58.4	3.45	26.44	severe drought
35	36.66	95.07	2701	Inland salt soil	*Folium Apocyni Veneti*	36.02	5.14	45.72	severe drought

**Table 2:** Effects of soil, plant nutrients and environmental factors on ecosystem stability in the optimal model of multiple linear regression. Note: Leaf total nitrogen (LTN), total phosphorus (LTP), total potassium (LTK), total carbon (LTC). Soil total nitrogen (STN), total phosphorus (STP), total potassium (STK), available phosphorus (AP), alkali-hydrolyzed nitrogen (AHN), soil stoichiometric ratios of carbon and nitrogen (Soil C:N), mean annual precipitation (MAP), mean annual temperature (MAT), soil soluble salts (SS), soil water content (SWC), d.f., the degree of freedom; SS, sum of squares; F, variance ratio; *p*, significance level; η^2^, Eta squared, the percentage of sum squares explained.

	d.f.	SS	F	*p*	η^2^ (%)
Stability, R^2^ = 0.47					
LTN	1	2.13	4.03	0.06	6.46
LTP	1	0.52	0.99	0.33	1.58
LTK	1	1.91	3.62	0.07	5.79
LTC	1	4.95	9.36	<0.01	15.00
STP	1	0.00	0.01	0.94	0.01
STK	1	0.01	0.02	0.89	0.03
STN	1	6.25	11.82	<0.01	18.92
MAT	1	2.47	4.67	<0.05	7.48
AP	1	2.49	4.71	<0.05	7.54
AHN	1	1.86	3.52	0.08	5.64
Soil C:N	1	0.01	0.01	0.92	0.02
SWC	1	0.49	0.93	0.35	1.49
SS	1	0.02	0.04	0.84	0.07
MAP	1	0.36	0.67	0.42	1.08
pH	1	0.03	0.05	0.83	0.08

## Data Availability

The data presented in this study are available on request from the corresponding author.

## Supplementary Materials

The following supporting information can be downloaded at: https://www.mdpi.com/article/10.3390/plants13131849/s1, Figure S1: Desert vegetation landscape in the Qaidam Basin. *Haloxylon ammodendron* community (A), *Salsola abrotanoides* community (B), *Sympegma regelii* community (C), *Achnatherum splendens* community (D); Figure S2: Variation trend in soil nutrient elements with MAT, MAP and AI; Figure S3: Variation trend in plant nutrient elements with MAT, MAP and AI; Figure S4: RDA of plant leaf nutrients, soil nutrients and environmental factors; Figure S5: NDVI change trend characteristics (A) and significance (B) in the Qaidam Basin from 2000 to 2020; Table S1: Area statistics of vegetation NDVI change trend in the Qaidam Basin from 2000 to 2020.

## References

[1] Feng S., Gu X., Luo S., Liu R., Gulakhmadov A., Slater L.J., Li J., Zhang X., Kong D. (2022). Greenhouse gas emissions drive global dryland expansion but not spatial patterns of change in aridification. J. Clim..

[2] Zeng H., Wu B., Zhang M., Zhang N., Elnashar A., Zhu L., Zhu W., Wu F., Yan N., Liu W. (2021). Dryland ecosystem dynamic change and its drivers in mediterranean region. Curr. Opin. Environ. Sustain..

[3] Berdugo M., Delgado-Baquerizo M., Soliveres S., Hernández-Clemente R., Zhao Y., Gaitán J.J., Gross N., Saiz H., Maire V., Lehmann A. (2020). Global ecosystem thresholds driven by aridity. Science.

[4] Maestre F.T., Eldridge D.J., Soliveres S., Kéfi S., Delgado-Baquerizo M., Bowker M.A., García-Palacios P., Gaitán J., Gallardo A., Lázaro R. (2016). Structure and functioning of dryland ecosystems in a changing world. Annu. Rev. Ecol. Evol. Syst..

[5] Báez S., Collins S.L., Pockman W.T., Johnson J.E., Small E.E. (2013). Effects of experimental rainfall manipulations on chihuahuan desert grassland and shrubland plant communities. Oecologia.

[6] Wu P., Zhou H., Cui Y., Zhao W., Hou Y., Tan C., Yang G., Ding F. (2022). Stoichiometric characteristics of leaf, litter and soil during vegetation succession in maolan national nature reserve, guizhou, china. Sustainability.

[7] Pugnaire F.I., Morillo J.A., Penuelas J., Reich P.B., Bardgett R.D., Gaxiola A., Wardle D.A., van der Putten W.H. (2019). Climate change effects on plant-soil feedbacks and consequences for biodiversity and functioning of terrestrial ecosystems. Sci. Adv..

[8] Tian H., Chen G., Zhang C., Melillo J.M., Hall C.A.S. (2009). Pattern and variation of c:N:P ratios in china’s soils: A synthesis of observational data. Biogeochemistry.

[9] Fan B., Westerband A.C., Wright I.J., Gao P., Ding N., Ai D., Tian T., Zhao X., Sun K. (2023). Shifts in plant resource use strategies across climate and soil gradients in dryland steppe communities. Plant Soil.

[10] Reich P.B., Oleksyn J. (2004). Global patterns of plant leaf n and p in relation to temperature and latitude. Proc. Natl. Acad. Sci. USA.

[11] Chen Y., Han W., Tang L., Tang Z., Fang J. (2013). Leaf nitrogen and phosphorus concentrations of woody plants differ in responses to climate, soil and plant growth form. Ecography.

[12] He M., Dijkstra F.A., Zhang K., Li X., Tan H., Gao Y., Li G. (2014). Leaf nitrogen and phosphorus of temperate desert plants in response to climate and soil nutrient availability. Sci. Rep..

[13] Zhang B., Tang G., Luo H., Yin H., Zhang Z., Xue J., Huang C., Lu Y., Shareef M., Gao X. (2021). Topsoil nutrients drive leaf carbon and nitrogen concentrations of a desert phreatophyte in habitats with different shallow groundwater depths. Water.

[14] Xu H.P., Zhang J., Pang X.P., Wang Q., Zhang W.N., Wang J., Guo Z.G. (2019). Responses of plant productivity and soil nutrient concentrations to different alpine grassland degradation levels. Environ. Monit. Assess..

[15] Wang X., Wang R., Gao J. (2022). Precipitation and soil nutrients determine the spatial variability of grassland productivity at large scales in china. Front. Plant Sci..

[16] Booth M.S., Stark J.M., Rastetter E. (2005). Controls on nitrogen cycling in terrestrial ecosystems: A synthetic analysis of literature data. Ecol. Monogr..

[17] Lambers H., Raven J.A., Shaver G.R., Smith S.E. (2008). Plant nutrient-acquisition strategies change with soil age. Trends Ecol. Evol..

[18] Fujita Y., van Bodegom P.M., Witte J.-P.M. (2014). Relationships between nutrient-related plant traits and combinations of soil n and p fertility measures. PLoS ONE.

[19] Li R., Wu P.-P., Peng C., Shi F.-X., Mao R. (2024). Shifted plant composition predominantly controls nitrogen addition effect on community-level leaf nutrient resorption in a boreal peatland. Plant Soil.

[20] Huang M.J., de Vries J., Zhou S., Hautier Y. (2023). Aridity and soil fertility, not species richness, interact to affect temporal stability of primary productivity along a natural gradient in northern china. Oikos.

[21] Li T., Kamran M., Chang S., Peng Z., Wang Z., Ran L., Jiang W.Q., Jin Y., Zhang X., You Y. (2022). Climate-soil interactions improve the stability of grassland ecosystem by driving alpine plant diversity. Ecol. Indic..

[22] Liu L., Zhu K., Wurzburger N., Zhang J. (2020). Relationships between plant diversity and soil microbial diversity vary across taxonomic groups and spatial scales. Ecosphere.

[23] Tognetti P.M., Prober S.M., Báez S., Chaneton E.J., Firn J., Risch A.C., Schuetz M., Simonsen A.K., Yahdjian L., Borer E.T. (2021). Negative effects of nitrogen override positive effects of phosphorus on grassland legumes worldwide. Proc. Natl. Acad. Sci. USA.

[24] Wang Y., Jiang L., Wang Z., Song M., Wang S. (2022). Phosphorus enrichment increased community stability by increasing asynchrony and dominant species stability in alpine meadow of qinghai-tibet plateau. J. Geophys. Res.-Biogeosci..

[25] Xu Q., Yang X., Yan Y., Wang S., Loreau M., Jiang L. (2021). Consistently positive effect of species diversity on ecosystem, but not population, temporal stability. Ecol. Lett..

[26] Orwin K.H., Dickie I.A., Wood J.R., Bonner K.I., Holdaway R.J. (2016). Soil microbial community structure explains the resistance of respiration to a dry–rewet cycle, but not soil functioning under static conditions. Funct. Ecol..

[27] Wang X., Yang M., Liang X., Pang G., Wan G., Chen X., Luo X. (2014). The dramatic climate warming in the qaidam basin, northeastern tibetan plateau, during 1961–2010. Int. J. Climatol..

[28] Zeng F., Zhang X., Zhan T., Zhang Z., Chen L., Chen L., Ji M. (2024). Rapid warming and increasing moisture levels in the qaidam basin. Theor. Appl. Climatol..

[29] Naeem S., Li S. (1997). Biodiversity enhances ecosystem reliability. Nature.

[30] Niu W., Chen H., Wu J. (2021). Soil moisture and soluble salt content dominate changes in foliar δ^13^c and δ^15^n of desert communities in the qaidam basin, qinghai-tibetan plateau. Front. Plant Sci..

[31] Kang Y., Guo E., Wang Y., Bao Y., Bao Y., Mandula N. (2021). Monitoring vegetation change and its potential drivers in inner mongolia from 2000 to 2019. Remote Sens..

[32] Zhang Y., He N., Loreau M., Pan Q., Han X. (2018). Scale dependence of the diversity-stability relationship in a temperate grassland. J. Ecol..

[33] Allen C.D., Macalady A.K., Chenchouni H., Bachelet D., McDowell N., Vennetier M., Kitzberger T., Rigling A., Breshears D.D., Hogg E.H. (2010). A global overview of drought and heat-induced tree mortality reveals emerging climate change risks for forests. For. Ecol. Manag..

[34] Tan Q., Jia Y., Wang G. (2021). Decoupling of soil nitrogen and phosphorus dynamics along a temperature gradient on the qinghai-tibetan plateau. Geoderma.

[35] Woods H.A., Makino W., Cotner J.B., Hobbie S.E., Harrison J.F., Acharya K., Elser J.J. (2003). Temperature and the chemical composition of poikilothermic organisms. Funct. Ecol..

[36] Chen S., Zhang W., Ge X., Zheng X., Zhou X., Ding H., Zhang A. (2023). Response of plant and soil n, p, and n:P stoichiometry to n addition in china: A meta-analysis. Plants.

[37] Joly F.-X., Kurupas K.L., Throop H.L. (2017). Pulse frequency and soil-litter mixing alter the control of cumulative precipitation over litter decomposition. Ecology.

[38] Delgado-Baquerizo M., Maestre F.T., Gallardo A., Bowker M.A., Wallenstein M.D., Quero J.L., Ochoa V., Gozalo B., García-Gómez M., Soliveres S. (2013). Decoupling of soil nutrient cycles as a function of aridity in global drylands. Nature.

[39] Luo Y., Peng Q., Li K., Gong Y., Liu Y., Han W. (2021). Patterns of nitrogen and phosphorus stoichiometry among leaf, stem and root of desert plants and responses to climate and soil factors in xinjiang, china. Catena.

[40] Hedin L.O. (2004). Global organization of terrestrial plant–nutrient interactions. Proc. Natl. Acad. Sci. USA.

[41] Sardans J., Peñuelas J. (2013). Tree growth changes with climate and forest type are associated with relative allocation of nutrients, especially phosphorus, to leaves and wood. Glob. Ecol. Biogeogr..

[42] Heineman K.D., Turner B.L., Dalling J.W. (2016). Variation in wood nutrients along a tropical soil fertility gradient. New Phytol..

[43] Andersen K.M., Corre M.D., Turner B.L., Dalling J.W. (2010). Plant–soil associations in a lower montane tropical forest: Physiological acclimation and herbivore-mediated responses to nitrogen addition. Funct. Ecol..

[44] Han W.X., Fang J.Y., Guo D.L., Zhang Y. (2005). Leaf nitrogen and phosphorus stoichiometry across 753 terrestrial plant species in china. New Phytol..

[45] Hayes P., Turner B.L., Lambers H., Laliberte E. (2014). Foliar nutrient concentrations and resorption efficiency in plants of contrasting nutrient-acquisition strategies along a 2-million-year dune chronosequence. J. Ecol..

[46] Luo W., Li M.-H., Sardans J., Lü X.-T., Wang C., Peñuelas J., Wang Z., Han X.-G., Jiang Y. (2017). Carbon and nitrogen allocation shifts in plants and soils along aridity and fertility gradients in grasslands of china. Ecol. Evol..

[47] Zhang B., Gao X., Li L., Lu Y., Shareef M., Huang C., Liu G., Gui D., Zeng F. (2018). Groundwater depth affects phosphorus but not carbon and nitrogen concentrations of a desert phreatophyte in northwest china. Front. Plant Sci..

[48] Yin H., Zheng H., Zhang B., Tariq A., Lv G., Zeng F., Graciano C. (2021). Stoichiometry of c:N:P in the roots of alhagi sparsifolia is more sensitive to soil nutrients than aboveground organs. Front. Plant Sci..

[49] Schimel D. (2004). Nutrient cycling and limitation: Hawai’i as a model system. Nature.

[50] He M., Zhang K., Tan H., Hu R., Su J., Wang J., Huang L., Zhang Y., Li X. (2015). Nutrient levels within leaves, stems, and roots of the xeric species reaumuria soongorica in relation to geographical, climatic, and soil conditions. Ecol. Evol..

[51] Tian Z. (2021). Relationship of Main Nutrient Elements in Soil and Vegetation in Qaidam Basin. Master’s Thesis.

[52] Wang L., Zhao G., Li M., Zhang M., Zhang L., Zhang X., An L., Xu S. (2015). C:N:P stoichiometry and leaf traits of halophytes in an arid saline environment, northwest china. PLoS ONE.

[53] Zhao G.S., Shi P.L., Wu J.S., Xiong D.P., Zong N., Zhang X.Z. (2017). Foliar nutrient resorption patterns of four functional plants along a precipitation gradient on the tibetan changtang plateau. Ecol. Evol..

[54] Shen Y., Yang X., Sun X., Chen W.Q., Yang G.W., Liu N., Chen J.S., Zhang Y.J. (2018). Increased precipitation modulates the influence of nitrogen and litter inputs on the nutrient resorption proficiency rather than efficiency of leymus chinensis. Plant Ecol..

[55] Glenn E.P., Brown J.J., Blumwald E. (1999). Salt tolerance and crop potential of halophytes. Crit. Rev. Plant Sci..

[56] Pessarakli M., Harivandi M.A., Kopec D.M., Ray D.T. (2012). Growth responses and nitrogen uptake by saltgrass (*Distichlis spicata* L.), a halophytic plant species, under salt stress, using the ^15^n technique. Int. J. Agron..

[57] Rahnama A., James R.A., Poustini K., Munns R. (2010). Stomatal conductance as a screen for osmotic stress tolerance in durum wheat growing in saline soil. Funct. Plant Biol..

[58] Huang K., Xia J. (2019). High ecosystem stability of evergreen broadleaf forests under severe droughts. Glob. Chang. Biol..

[59] Liu P., Chi Y., Chen J., Zhou L. (2023). Global climate regulates dimensions of terrestrial ecosystem stability. Ecosphere.

[60] Yu H., Ma Q., Liu X., Li Y., Li L., Qi M., Wu W., Wang Y., Xu Z., Zhou G. (2021). Resistance, recovery, and resilience of desert steppe to precipitation alterations with nitrogen deposition. J. Clean. Prod..

[61] Chen B., Chen H., Li M., Fiedler S., Mărgărint M.C., Nowak A., Wesche K., Tietjen B., Wu J. (2022). Climate sensitivity of the arid scrublands on the tibetan plateau mediated by plant nutrient traits and soil nutrient availability. Remote Sens..

[62] Balazs K.R., Munson S.M., Butterfield B.J. (2022). Functional composition of plant communities mediates biomass effects on ecosystem service recovery across an experimental dryland restoration network. Funct. Ecol..

[63] Song M.-H., Zong N., Jiang J., Shi P.-L., Zhang X.-Z., Gao J.-Q., Zhou H.-K., Li Y.-K., Loreau M. (2019). Nutrient-induced shifts of dominant species reduce ecosystem stability via increases in species synchrony and population variability. Sci. Total Environ..

[64] Liu J., Li X., Ma Q., Zhang X., Chen Y., Isbell F., Wang D. (2019). Nitrogen addition reduced ecosystem stability regardless of its impacts on plant diversity. J. Ecol..

[65] Zhang T., Ji X., Xu M., Zhao G., Zheng Z., Tang Y., Chen N., Zhu J., He Y., Zhang Y. (2022). Influences of drought on the stability of an alpine meadow ecosystem. Ecosyst. Health Sustain..

[66] Liu X., Ma Q., Yu H., Li Y., Li L., Qi M., Wu W., Zhang F., Wang Y., Zhou G. (2021). Climate warming-induced drought constrains vegetation productivity by weakening the temporal stability of the plant community in an arid grassland ecosystem. Agric. For. Meteorol..

[67] Gao Q., Ganjurjav H., Hu G., Xu H., Schwartz M.W., Gornish E.S., Zhu W. (2022). Warming diminishes the stability of primary productivity in global grass- and forb-dominated ecosystems. Environ. Res. Commun..

[68] Shi Z., Xu X., Souza L., Wilcox K., Jiang L., Liang J., Xia J., García-Palacios P., Luo Y. (2016). Dual mechanisms regulate ecosystem stability under decade-long warming and hay harvest. Nat. Commun..

[69] Zhang L., Xiao P., Yu H., Zhao T., Liu S., Yang L., He Y., Luo Y., Wang X., Dong W. (2022). Effects of climate changes on the pasture productivity from 1961 to 2016 in sichuan yellow river source, qinghai-tibet plateau, china. Front. Ecol. Evol..

[70] Gao Y., Zhou X., Wang Q., Wang C., Zhan Z., Chen L., Yan J., Qu R. (2013). Vegetation net primary productivity and its response to climate change during 2001–2008 in the tibetan plateau. Sci. Total Environ..

