# Supplementary material for: In the Qaidam Basin, Soil Nutrients Directly or Indirectly Affect Desert Ecosystem Stability under Drought Stress through Plant Nutrients
Source: Plants (Basel). 2024 Jul 5;13(13):1849. doi: 10.3390/plants13131849 (PMC11244565; doi:10.3390/plants13131849)
Supplement: Supplementary file 1 [file plants-13-01849-s001.zip › plants-3052067-supplementary.pdf]

In the Qaidam Basin, Soil Nutrients Directly or Indirectly Affect Desert Ecosystem Stability under Drought Stress through Plant Nutrients

Yunhao Zhao, Hui Chen*, Hongyan Sun and Fan Yang

Hebei Key Laboratory of Environmental Change and Ecological Construction,
Hebei Technology Innovation Center for Remote Sensing Identification of Environmental Change,
School of Geographical Sciences, Hebei Normal University, Shijiazhuang 050024, China
* Correspondence: chenhui@hebtu.edu.cn

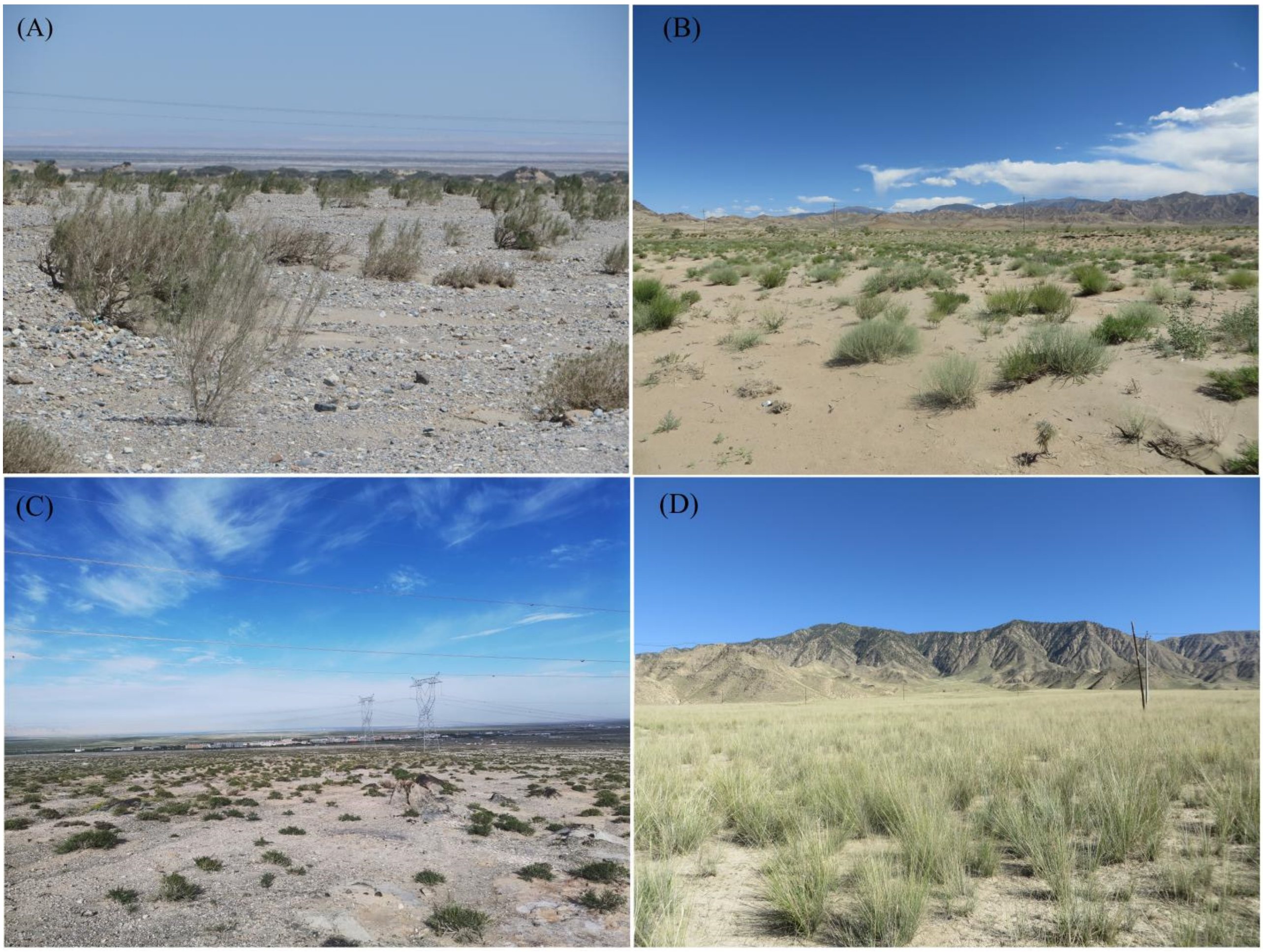

Figure S1. Desert vegetation landscape in Qaidam basin. *Haloxylon ammodendron* community (A), *Salsola abrotanoides* community (B), *Sympegma regelii* community (C), *Achnatherum splendens* community (D).

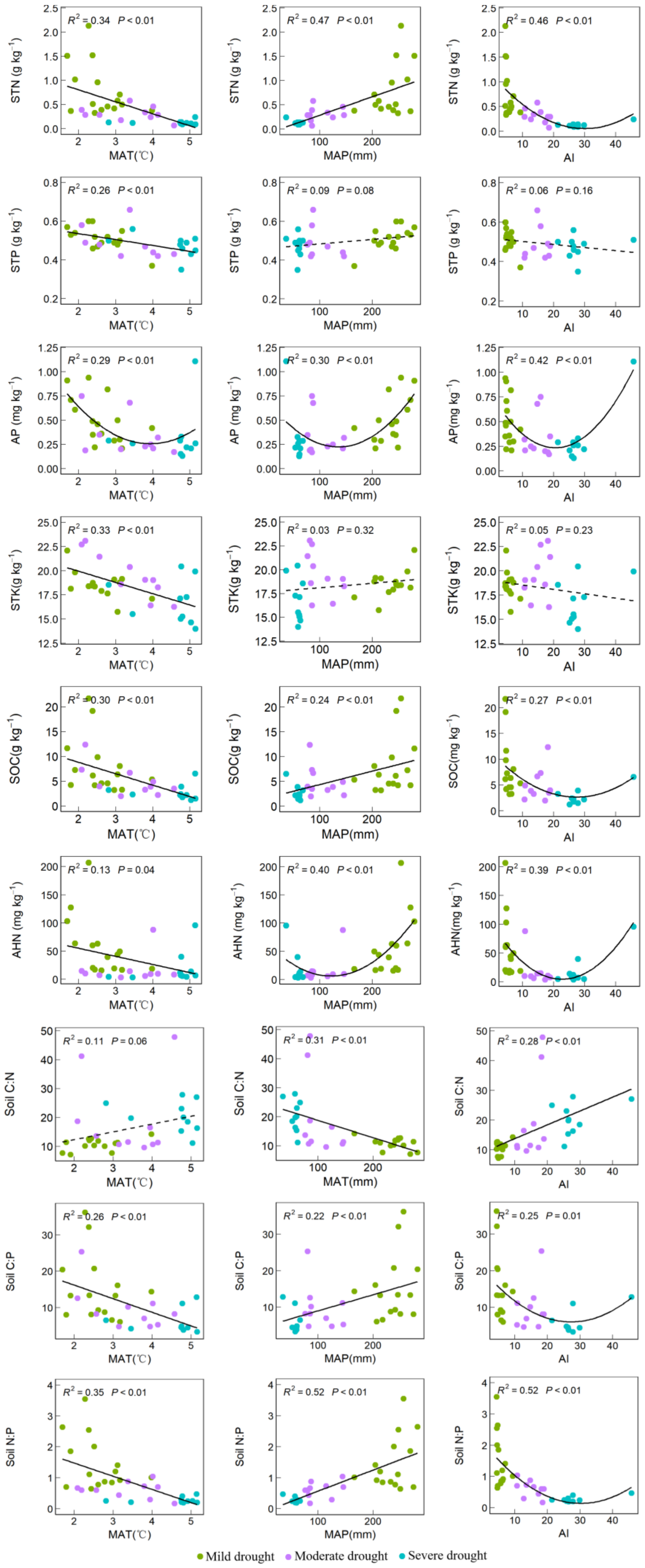

Figure S2. Variation trend in soil nutrient elements with MAT, MAP and AI.

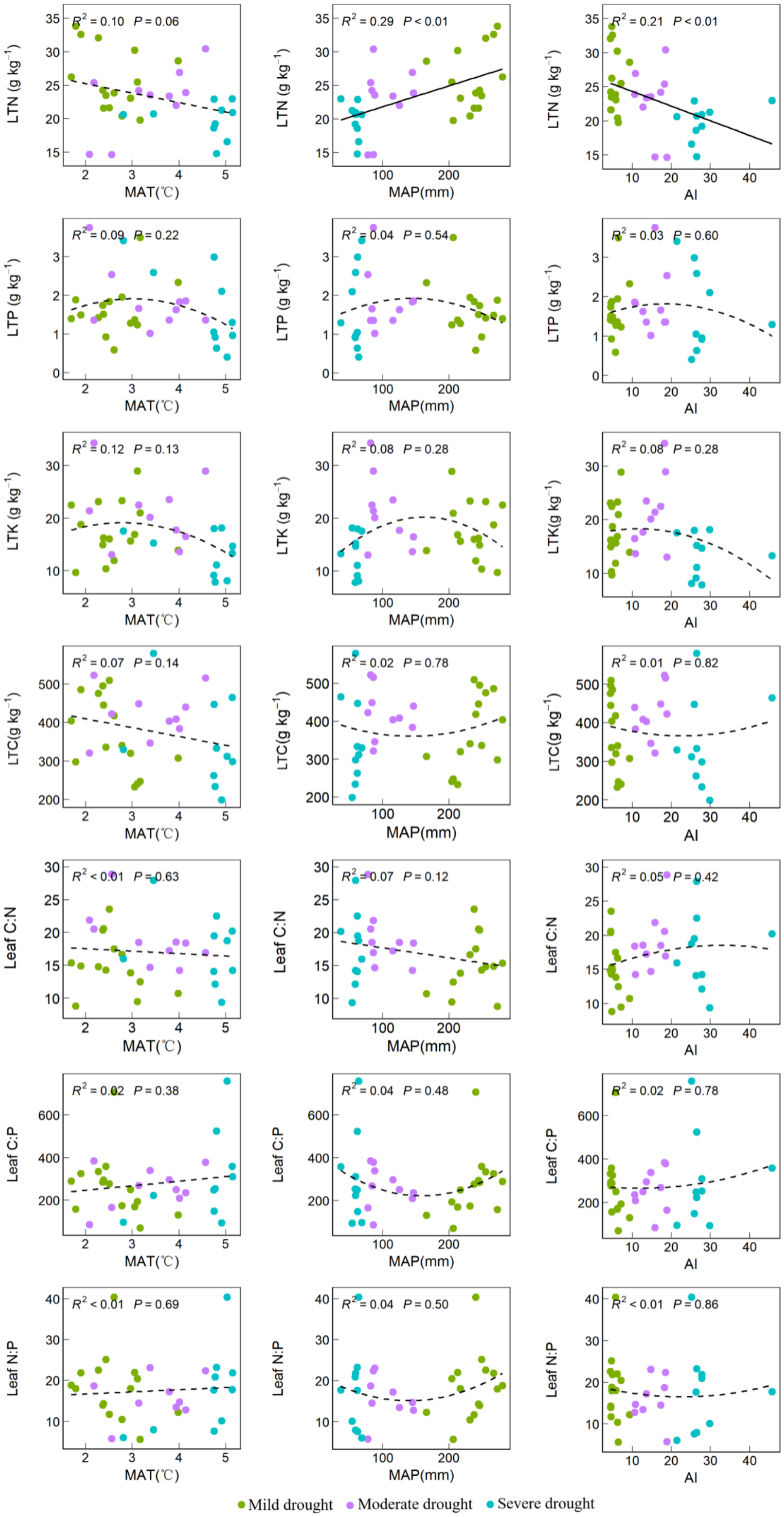

Figure S3. Variation trend in plant nutrient elements with MAT, MAP and AI.

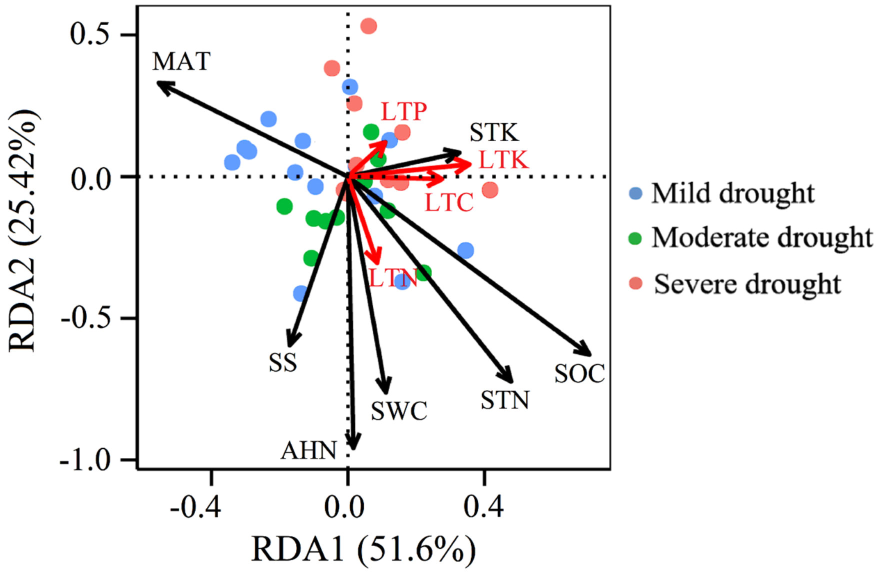

Figure S4. RDA of plant leaf nutrients, soil nutrients and environmental factors.
Note: Environmental factors include soil soluble salts (SS), soil water content (SWC), mean annual temperature (MAT), and soil and leaf nutrient abbreviation as above.

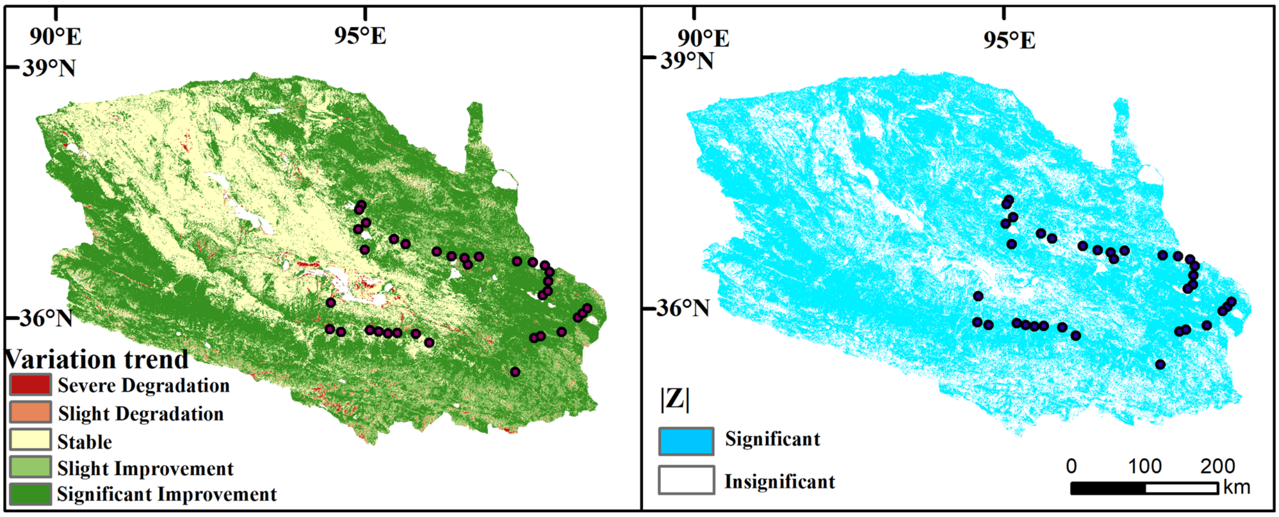

Figure S5. NDVI change trend characteristics (A) and significance (B) in Qaidam Basin from 2000 to 2020.

Table S1.
Area statistics of vegetation NDVI change trend in Qaidam Basin from 2000 to 2020.

Vegetation NDVI change trend	Area Percentage /%
Severe degradation	0.66%
Slight degradation	1.36%
Stable	33.23%
Slight improvement	14.52%
Significant improvement	50.24%
